# The Cultural Adaptation of the Everyday Problems Test—Greek Version: An Instrument to Examine Everyday Functioning

**DOI:** 10.1177/23337214211027683

**Published:** 2021-07-05

**Authors:** George Pavlidis, Stephanie Hatzifilalithis, Nikolaos Marwan Zawaher, Georgia Papaioannou, Eleni Giagkousiklidou, Ana B. Vivas

**Affiliations:** 1 Linköping University, Norrköping, Sweden; 2 McMasters University, Hamilton, ON, Canada; 3 The University of Sheffield, International Faculty, Thessaloniki, Greece

**Keywords:** ageing, cognitive decline, everyday functioning, IADL

## Abstract

Assessing cognitive decline and everyday functioning (EvF) in older age is valuable in detecting age-related neurological disorders. In Greece, there is a lack of sensitive instruments that capture fluctuations in EvF among older persons who are cognitively healthy or have subtle cognitive impairments. The EPT 28-items test, a widely used paper-and-pencil EvF measure, was translated in Greek and adapted to the Greek culture in this study. A multi-step methodology using a sample of 139 older Greek persons was employed. The results indicate that the Greek version of the EPT 28-items (i.e., the EPT-G) was well adapted, representing everyday tasks in Greece within a good range of task difficulty. The psychometric properties of the EPT-G replicate those of the original instrument, capturing EvF fluctuations among older persons with mild cognitive impairments. It was concluded that the EPT-G is a useful measure of EvF among Greek older persons.

## Introduction

There is growing interest in examining the cognitive and functional abilities of older people (Schmitter-Edgecombe & Farias, 2018; Tomaszewski-Farias et al., 2018). The evaluation frameworks on autonomy, dependency, and care needs in older age highlight the need to understand the timepoint at which older persons’ cognitive capacity fails to sustain their everyday functioning (van Harten et al., 2018). The relevant scientific evidence shows that impaired cognitive performance (CP) has adverse effects on the ability to carry out everyday tasks and live autonomously (Hering et al., 2018). Cognitive impairments among older persons are the main reason for referrals for neuropsychological assessment, with potential outcomes including a diagnosis of mild cognitive impairment (MCI), dementia, or other age-related neurodegenerative disorders (Larner, 2018; Marcotte et al., 2010). The evaluation of older adults’ abilities to deal independently with everyday tasks has been predominately carried out via assessments of activities of daily living (ADL) and instrumental activities of daily living (IADL; Moore et al., 2007; Scheel-Hincke et al., 2020). A valid measure of everyday functioning, along with neuropsychological tests of executive function, memory, and speed of processing, are essential and complementary parts of a diagnosis of MCI or dementia (Petersen, 2004; Petersen et al., 2018; Ramanan et al., 2017).

Recent meta-analyses of studies examining older adults diagnosed with MCI and dementia indicate a moderate relationship between CP assessments and measures of everyday functioning (EvF). Among the neuropsychological assessments, measures of executive function (EF) seem to have the strongest correlations with EvF (Lindbergh et al., 2016; Martyr & Clare, 2012; Mcalister et al., 2016; Royall et al., 2007). Evidence regarding the relationship between CP and EvF in cognitively intact older adults is sparse. This may reflect a lack of interest in examining the potential of healthy older adults, and an increased interest in scrutinizing disability in older age (Peel et al., 2004). As noted by Kelly et al. (2014), it is often the case that clinical trial studies do not include EvF measures to assess the cognitive benefits of cognitive training interventions on everyday functioning, particularly when cognitively healthy older adults are studied. However, *research on EvF among older adults continues to gain prominence, as the identification of subtle decrements may facilitate early diagnosis of age-related cognitive impairments. This creates a need to develop and validate sensitive tools that can detect subtle decrements in EvF among cognitively healthy older adults* (Peel et al., 2004), or among those who maintain a high level of everyday functioning despite mild cognitive impairments.

Cherner (2010) pointed out that the assessment of EvF largely depends on the cultural relevance of the available tools. In Greece, the available tools for assessing EvF are mostly questionnaire-based ADL measures, such as the Greek version of the Lawton and Brody Scale (Vlachos et al., 2020). Diehl et al. (2005) argued that questionnaire-based assessments of EvF are susceptible to over-estimation or under-estimation biases of actual behaviors. Therefore, task-oriented EvF tests may provide a useful alternative for assessing EvF among older adults. The Functional Cognitive Assessment Scale (FUCAS) is a task-oriented EvF assessment for older Greek adults, with psychometric properties that can detect MCI in dementia patients with severe cognitive impairments (Kounti et al., 2006). However, this measure may lack the necessary sensitivity to capture subtle deteriorations in EvF in cognitively healthy older adults, or in those who maintain their functional independency despite subtle cognitive impairments. Some tools available in English have been suggested to have the necessary sensitivity to capture EvF decrements caused by subtle cognitive deficiencies, one of them being the Everyday Problems Test (Moore et al., 2007; Willis et al., 2006). The Everyday Problems Test (EPT) is available free of charge and features good psychometric properties and detailed administration instructions (Moore et al., 2007).

The EPT is a paper-and-pencil test in which the examinees are expected to solve and respond to everyday tasks of shopping, telephone use, driving, medication handling, finance handling, nutrition, and commuting. Over the years, four versions of the test have been developed: the 84-item and 48-item versions by Willis (1990), the 32-item version (Willis, 1993), and the 28-item version (Willis et al., 2006). The last version was developed in the context of the ACTIVE study to shorten the administration time of testing (Kimbler, 2013). Performance in the EPT 28-items has been weakly correlated with scores in the questionnaire-based IADL measure of Lawton and Brody (1969), and strongly correlated with the task-oriented assessment known as the Observed Tasks of Daily Living (Diehl et al., 1995). In the original study of the EPT 28-items version (Kimbler, 2013), performance in the EPT 28-items measure correlated significantly with neuropsychological assessments of inductive reasoning (the Letter Series Test, *r* = .61) and age (*r* = −.38), but not with verbal ability (the Verbal Meaning Test). The correlation of EPT 28-items with education, as well as its test-retest reliability and internal consistency, were not reported in Kimbler (2013). However, for the 32-items of the EPT measure the 2-month test-retest reliability index was *r* = .94, the internal consistency was high (*a* = 0.84), and the total score had a significant correlation (*r* = .49) with education (Willis, 1993).

The cost-free availability of the tool, the good psychometric properties, and its simple modus of administration makes the EPT 28-items version a good solution for clinicians and researchers examining EvF (see adaptation of the EPT in the Italian context from Cantarella et al., 2017). A visual inspection of the everyday tasks in the EPT 28-items reveals important cultural dependencies on the sociocultural context of the USA, where the instrument was developed. For example, the metrics used (i.e., dollars for currency, feet for distance) are distinct from those used in Greece (i.e., euros for currency, meters for distance), whereas balancing a checking account or writing a check to pay an electricity bill by post is an unfamiliar task to most Greek older persons. To provide a sensitive instrument that allows the examination of EvF among older adults in Greece, these discrepancies were addressed in this study to make the Greek version a context-relevant instrument. This study therefore was set out to translate, culturally adapt, and evaluate the psychometric properties of a Greek version of the EPT 28-items test.

## Methods

Approval for using and translating of the original EPT-28items tool has been obtained. In the absence of specific guides for the cultural adaptation of EvF measures, the translation and adaptation process of the EPT 28-items followed the recommendations of Geisinger (1994) on adapting an assessment instrument to a new culture and language population. A multi-step approach following seven steps was followed, consisting of: Adapt and translate the measure; Review the translated and adapted versions of the instruments; Adapt the draft instruments based on the comments of the reviewers; Pilot testing of the instruments; Field testing of the instrument and proceed to validation research; Develop manuals and other documents for the users of the assessment device; Train users—collect reactions from users. The study was approved by the University Research Ethics Committee of the University of (BLINDED). All participants signed an informed consent form.

### Participants

For the purposes of the study, 154 adults over the age of 50 were recruited through poster announcements at the university and at three senior citizens’ centers in Thessaloniki, Greece. Of the total, 15 participants were excluded based on predetermined criteria: 4 because Greek was not their native language; 6 had a stroke, seizures, depression, or Parkinson’s; 3 were taking psychoactive medication (sedatives) or were under the influence of alcohol at the time of testing; and 2 had dyslexia or were illiterate.

### Instruments

#### Mini mental state examination (MMSE)

The Greek version of the MMSE was used in the screening process. The MMSE is a broadly used instrument to screen for general cognitive deficits, assisting with the detection of dementia (Fountoulakis et al., 2000). The MMSE is standardized for the Greek population and has a cut off score of 23, with scores under this threshold indicating serious neuropsychological impairments consistent with the existence of various forms of dementia.

#### Montreal cognitive assessment (MoCA)

The Greek version of the Montreal Cognitive Assessment (MoCA) was used to assess whether participants exhibit symptoms that are consistent with a diagnosis of MCI. The normative data for the Greek older population for the MoCA is available in Poptsi et al. (2019), with the cut-off scores being 23 for those with a lower educational level (≤6 years), and 26 for those with a higher educational level (≥7 years).

#### Verbal fluency

Verbal fluency (VF) was measured by asking the participants to recall as many words that start with the Greek letter “*x*” as possible within 1 minute. This item derives from the MoCA instrument, and is used as an assessment of verbal fluency in the Survey of Health and Retirement in Europe (SHARE) study (Aichberger et al., 2010).

#### Trail making test parts A and B

The Greek versions of the Trail Making Test Part A (TMT-A) and the Trail Making Test Part B (TMT-B) (Vlachou & Kosmidou, 2002) were administered to all participants. TMT-A is a timed test that requires participants to draw a line connecting 25 consecutive numbers. TMT-B is timed and requires participants to connect consecutive numbers and letters, alternating between the two sequences (i.e., 1-A-2-B-3-C . . . L-12). During the administration of the TMT-A and TMT-B, participants’ errors were pointed out by the researchers. The participants were redirected to the last correct circle while timing continued. Two indices were calculated: (i) the seconds required to complete the TMT-A task, as an indicator of speed of processing, and (ii) the seconds required to complete the TMT-B task. These indices have been frequently used as indices of cognitive flexibility and set-shifting (Hester et al., 2005). Therefore, higher scores in TMT-A and TMT-B indicate poorer executive function (EF).

#### California verbal learning test

The Greek version of the California Verbal Learning Test (GVLT) is a verbal memory measure, which assesses learning, retention, and recall of verbal information (Vlachou et al., 2012). The GVLT is the Greek adaptation of the California Verbal Learning Test (CVLT), in which five learning trials are administered to the examinees (Delis et al., 1987). The researcher reads out loud a shopping list for Monday, and the participant is requested to recall as many of the items as possible after each reading. The interference (Tuesday) list was read subsequently, followed by an immediate free recall of the Monday list. Approximately 20 minutes later, the participants were asked to recall the items from the initial list. The indices of memory capacity were created following previous examples (Vlachou et al., 2012), and were: (i) the number of words remembered after the first reading of the Monday list, a measure of immediate recall (IR), (ii) the total number of words recalled during the five learning trials, a measure of the participants’ learning curve (LC), (iii) the total number of words recalled after the interference list, a measure of short-term memory (STM), and (iv) the number of words recalled after the 20 minutes interval, a measure of long-term memory (LTM).

#### Perceived health

Perceived health was assessed using the item “Would you say that your health is. . .,” with the available forced choices ranging from “excellent,” “very good,” “good,” “Fair,” to “poor.” As Rodin and McAvay (1992) demonstrated, self-reported health is closely related to actual health, as well as being independently predictive of chronic illnesses, sick days, hospitalization, and medication use, especially in seniors.

#### Education and income

Education was measured with an ordinal scale reflecting educational levels in Greece. Income was measured by the open-ended item “total amount of income at the end of the year.” The sum then was divided in 12 parts and included as an average monthly income in the analyses.

### Procedure for Translation and Adaptation

#### Step 1: Adaptation and translation of EPT 28-items

The metrics of the original EPT 28-items were replaced with meters (for distance) and euros (for currency). Names, telephone numbers, cities, and telecommunication providers were replaced with Greek ones. The eighth item in EPT 28-items was modified to represent taxi rates when traveling in a foreign country (i.e., in a hypothetical scenario of traveling abroad). The items were translated and back translated by two independent bilingual neuropsychologists, as recommended by Hambleton (2001), after the first author (BLINDED) had explained and clarified the concepts and meanings of the items to reduce the risk of misinterpretation, as recommended by Nordin et al. (2015). The scoring range and the calculation principles of the Greek version of the EPT 28-items remained unchanged, to preserve the scoring equivalence with the original measure.

#### Step 2: Revision of the translated and adapted versions of the Greek version of EPT 28-items

Two independent Greek-English bilingual neuropsychologists and an English-Greek bilingual sociologist with expertise in social gerontology checked for the conceptual equivalence of the adjusted items and the quality of the translation. The panel translating the EPT 28-items version shared their comments among themselves, as well as with the first author (BLINDED). No issues emerged from this discursive process. Given that the review panel did not raise any issues regarding the translation and the adaptation of the instruments, no further revisions were made. A Greek-English bilingual philologist of English literature and the English-Greek bilingual sociologist independently checked for the linguistic appropriateness of the items and the materials used in the adapted version of the EPT 28-items. They concluded that the final instruments reflect—to the best of their judgment—everyday tasks relevant to Greek older adults.

#### Step 3: Pilot testing of the instrument

The instrument was administered initially to the first 13 participants (5 belonging to the MCI group and 8 to the cognitively intact group) of the study to examine the understandability of the instructions, the acceptability of the time limits, and the wording of the items. According to Bujang and Baharum (2017), a sample size of 13 is recommended to reach an ICC average of 0.700 and over, with power = 90% and *p* < .05 for two observations per participant. Each participant underwent a 2-hour session on a one-to-one basis and after an interval of 2 weeks, the EPT-G was administered to them again. The participants were given the opportunity to comment on the items separately, as well as for the instrument, with no issues emerging in this respect. The only emerging theme was that of familiarity for some items (proceed with financial affairs, nutrition, cooking issues, and driving), which will be discussed below.

#### Step 4: Field testing of the instrument

The reliability of the EPT-G was assessed through internal consistency analysis and inter-rater reliability analysis. A comparison of the EPT-G with the original instrument was carried out as suggested by Geisinger (1994). Test re-test reliability analysis was conducted only in the initial subsample (*N* = 13) of older adults.

#### Step 5: Validation process

All participants, including the initial subsample of the participants, completed the testing protocol, including demographics, neuropsychological assessments, and the EPT-G. Following Geisinger’s (1994) suggestion, a criterion-related validity was used to test the validity of the EPT-G as an instrument that assesses EvF. The concurrent criterion-related validation consists of the test’s score correlation with certain criteria. Based on the available literature on the validation of the original EPT 28-items and previous practice (Diehl et al., 2005), the authors opted for the instruments’ correlation with age, education, and basic cognitive abilities as the criteria of validity.

#### Step 6: Developing a manual for the users of the assessment

The original EPT 28-items is accompanied by a scoring manual produced for the purposes of the ACTIVE study. This guide was translated and adapted for the development and field testing of the EPT-G.

#### Step 7: Train users—collect reactions from users

The first author (BLINDED) engaged in a training process with all the other researchers involved in data collection. The training program consisted of (i) a review of the literature for the original instruments, (ii) a review of the training material available online for the original instruments, (iii) undertaking the adapted (i.e., the EPT-G) instruments as participants, (iv) administering the tests to (BLINDED), (v) administering the tests to each other with the supervision of (BLINDED), (vi) administering the tests to older individuals under the supervision of (BLINDED), (vii) scoring three tests, subsequently reviewed by (BLINDED). Depending on the level of confidence expressed by the first author and the users administering the tests, each trainee underwent 5 to 15 hours of training. The trainees were able to comment on the instruments throughout the training process. Through a formal discursive process, their comments were discussed, and a final mutual agreement was reached for the implementation of the instrument in its final form. A table detailing the steps, processes, and outcomes of the translation and adaptation process from the EPT 28-items to the EPT-G is available in the Supplemental Appendix I.

### Procedure of Test Administration

Participants were examined by one of the authors in an individual basis in a quiet room, either in a laboratory in the University premises or in the examination rooms in the Senior Day Centers. Privacy and confidentiality of the process and of the results were preserved. The neuropsychological assessments’ administration followed standardized practices and their scoring followed the protocols and procedures described in the respective instruments’ manual.

### Data Analysis Plan

The quantitative data were analyzed using SPSS v.26. To examine the test-retest reliability of the instrument, intra-class correlation (ICC) analyses was conducted between the EPT-G scores administered to a subsample of the participants in two occasions, using a two-way random model examining absolute agreement between rating. The internal consistency of the EPT-G was examined though Cronbach’s alpha test in the whole sample. Frequency analysis was used to examine task difficulty levels of the EPT-G items in the whole sample. Similarly, one-way ANOVA analysis was conducted to examine differences in EPT-G scores, demographics, and scores in neuropsychological assessments between the cognitively intact, MCI group, and dementia group. Pearson correlation coefficients analyses was conducted to examine whether EPT-G scores replicates the associations of the original instrumental with age, education, and scores in neuropsychological assessments. For the same reason, linear regression analyses were conducted to examine independent predictors of EPT-G scores among the demographic variables (age, gender, income, and education), cognitive status, and scores in the neuropsychological assessments. Following the reporting of the original instrument (Willis, 1993), linear regression analyses stratified by cognitive status was conducted to examine independent predictors of EPT-G scores among the demographic variables (age, gender, income, and education), health, and scores in neuropsychological assessments.

According to Lumley et al. (2002), a sample size of 100 is sufficiently large for the normal distribution of the data to be rendered as unimportant, when the level of confidence is set to *p* = .05. In addition, simulation studies (Bujang et al., 2017; Knofczynski, 2017) have demonstrated that a sample size of 100 is sufficient for a good prediction level for regression models including nine or more independent predictors, when the targeted unadjusted variance explained by the models are set to be *R*^2^ = .50. Given these preconditions, the sample size of this study was rendered as sufficient.

## Results

From the 139 participants, seven participants had a Mini Mental State Examination (MMSE) score below 23, and thus might have been living with dementia (Fountoulakis et al., 2000). By applying the cut-off principles of the Montreal Cognitive Assessment (MoCA) scores in the Greek population (Poptsi et al., 2019), 69 participants were categorized as potentially having MCI. The remaining 63 participants were categorized as cognitively intact. The demographic information of the sample is presented in Table 1. None of the participants with suspected dementia was able to complete the EPT-G, whereas only one of them managed to complete the TMT-B test. Two participants in the dementia group did not manage to complete the TMT-A and one of them did not manage to complete the GVLT. From those with suspected MCI, 13% were unable to complete the EPT-G and 6% the TMT-B. For the participants in the MCI group who did not manage to complete the test, a score of 0 was assigned manually. All participants categorized as cognitively intact were able to complete the EPT-G and the neuropsychological measures.

**Table 1.** Demographics for the Whole Sample Divided by Cognitive Status.

	All (%)		Healthy (%)		MCI (%)		Dementia (%)	
Gender								
Men	35.3		31.7		36.2		57.1	
Women	64.7		68.3		63.8		42.9	
Education (level)								
None	1.4		0		1.4		14.3	
ISCED 1	15.8		7.9		18.8		57.1	
ISCED 2	5		3.2		7.2		0	
ISCED 3	35.3		36.5		37.7		0	
ISCED 4 and 5	14.4		15.9		14.5		0	
ISCED 6	24.5		34.9		14.5		28.6	
ISCED 7	3.6		1.6		5.8		0	
Perceived health								
Excellent	8.6		9.5		7.2		14.3	
Very good	25.9		25.4		27.5		14.3	
Good	36.7		33.3		37.7		57.1	
Fair	25.2		31.7		20.3		14.3	
Poor	3.6		0		7.2		0	
Marital status								
Never married	5.8		9.5		2.9		0	
Married or cohabiting	67.6		63.5		72.5		57.1	
Divorced or separated	9.4		15.9		4.3		42.9	
Widowed	17.3		11.1		20.3		0	
	*M*	*SD*	*M*	*SD*	*M*	*SD*	*M*	*SD*
Age	64.92	8.27	61.08	6.36	66.88	7.43	80.14	7.79
Income^a^	10.89	8.42	10.39	6.16	11.26	10.08	11.43	7.13

*Note*. ISCED = international standard classification of education.

a In hundred euros.

One-way ANOVA analysis revealed statistically significant differences between the cognitively intact, MCI, and dementia groups in terms of their average age (*F*[2, 138] = 28.861, *p* < .000), education (*F*[2, 138] = 5.682, *p* < .005), MMSE scores (*F*[2, 138] = 78.202, *p* < .000), MoCA scores (*F*[2, 138] = 135.457, *p* < .000), VF scores (*F*[2, 138] = 7.356, *p* < .005), IR scores (*F*[2, 137] = 14.167, *p* < .000), LC scores (*F*[2, 137] = 25.128, *p* < .000), STM scores (*F*[2, 137] = 27.102, *p* < .000), and LTM scores (*F*[2, 137] = 27.808, *p* < .000). Subsequent Tukey post-hoc comparison (see Table 2) indicated that the cognitively intact group was younger and had higher scores than the MCI group in almost all neuropsychological assessments, except in TMT-A. Similarly, subsequent Tukey post-hoc comparison indicated that the MCI group was younger and had higher scores than the dementia group in almost all neuropsychological assessments, except in TMT-A and VF.

**Table 2.** Mean and Standard Deviations of Demographics and Cognitive Scores, Along with Significance Values (*p*s) of Post-Hoc Tukey Analysis Identifying Statistically Significant Differences between Older People of Different Cognitive Status.

	Healthy		MCI		Dementia		Healthy MCI	MCI dementia
	*M*	*SD*	*M*	*SD*	*M*	*SD*	*p*s	*p*s
Age	61.08	6.36	66.88	7.43	80.14	7.79	.000	.000
Income^a^	10.39	6.16	11.26	10.08	11.43	7.13	.834	.999
Education^b^	4.71	1.24	4.14	1.55	3.00	2.08	.067	.120
Health^c^	2.87	.97	2.92	1.03	2.71	.95	.948	.854
MMSE	28.77	1.52	27.47	1.77	20.71	.95	.000	.000
MoCA	27.26	1.23	22.17	2.53	17.14	3.76	.000	.000
VF	13.31	3.60	11.25	3.90	9.00	4.04	.006	.294
TMT-A	50.74	46.40	48.92	18.59	86.40	33.54	.951	.055
TMT-B	137.25	101.13	116.55	99.91	156.66	99.76	.025	—
IR	6.89	2.52	5.93	1.70	2.33	.816	.026	.000
LC	51.35	11.31	41.57	12.20	21.17	4.49	.000	.000
STM	10.86	2.89	8.57	3.22	2.00	2.75	.000	.000
LTM	10.90	3.37	9.07	3.60	1.67	2.25	.008	.000
EPTscore	17.44	6.35	11.28	8.86	—	—	—	—
EPTtime	42.77	15.02	48.79	18.58	—	—	—	—

*Note*. MMSE = mini mental state examination; MoCA = montreal cognitive assessment; VF = verbal fluency; TMT = trail making test; IR = immediate recall; LC = learning curve; STM = short term memory; LTM = long term memory; EPT = everyday problem test.

a In hundred euros.

b Refers to a dummy variable created to represent educational levels.

c Refers to a dummy variable created to represent health status.

**Figure 1.**
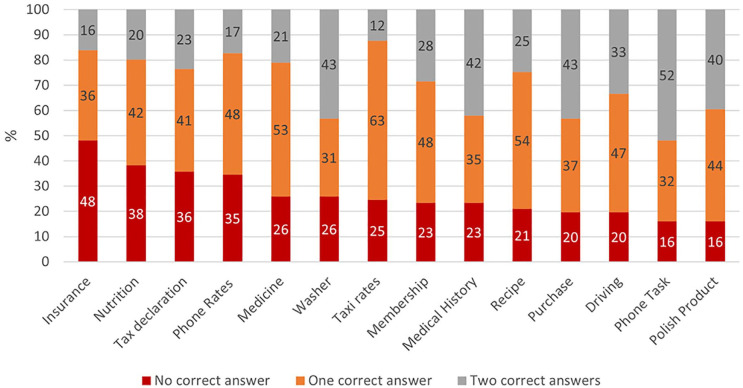
Percentage of correct responses in each of the 14 everyday tasks.

Intra-class correlation coefficients analyses in the EPT-G scores in a subsample consisting of eight cognitively intact and five potential MCI participants revealed an acceptable test-retest reliability ICC = 0.846, *p* = .001. The EPT-G had an internal consistency of α = .892, which is within acceptable levels. One-way ANOVA analysis did not reveal any statistically significant differences in EPT-G scores between those administering the test (*F*[6, 122] = 1.130, *p* = .350). In terms of the difficulty of the questions, a visual inspection of Figure 1 reveals that the EPT-G has difficulty levels ranging from easy questions (e.g., the phone task), where 52% of the participants answered both questions correctly, to more demanding questions (e.g., Insurance), where 48% had only incorrect responses.

The average completion time for the EPT-G was 45.71 minutes (*SD* = 17.05), and the mean score was 13.51 (*SD* = 7.64). Independent samples *t*-test revealed statistically significant differences in EPT-G scores (*t*[131] = −4.567, *p* < .000) but not in EPT time of completion (*t*[131] = 1.964, *p* > .05) between the cognitively intact and MCI groups. Pearson correlation coefficients analyses revealed statistically significant relationships of EPT-G scores with most of the demographics (except health) and neuropsychological assessments (see Table 3).

**Table 3.** Pearson Correlation Coefficients of Demographics with Performance in Neuropsychological Measures and the Everyday Problems Test—Greek Version Among the Cognitively Health and MCI Group.

	1	2	3	4	5	6	7	8	9	10	11	12	13	14
	EPT_score_	EPT_time_	Age	Income	Education^a^	Health^b^	MMSE	MoCA	VF	TMT-A	TMT-B	IR	LC	STM
2	.007													
3	−.508**	.159												
4	.200*	−.068	−.030											
5	.573**	−.068	−.321**	.278**										
6	−.089	.056	−.051	−.198*	−.130									
7	.455**	−.072	−.495**	.033	.268**	−.003								
8	.570**	−.113	−.542**	.016	.407**	.031	.611**							
9	.324**	.009	−.209*	.178*	.269**	−.050	.316**	.410**						
10	−.170*	.035	.113	.066	−0.126	−.052	−.274**	−0.158	−.108					
11	−.406**	.011	.418**	−.115	−.199*	.117	−.288**	−.335**	−.208*	.135				
12	.416**	−.125	−.338**	−.073	.248**	.010	.448**	.400**	.235**	−.144	−.246**			
13	.500**	−.295**	−.453**	−.036	.230**	.068	.445**	.557**	.214*	−.228**	−.262**	.620**		
14	.501**	−.192*	−.481**	−.163	.252**	.012	.436**	.538**	.224**	−.107	−.263**	.621**	.741**	
LTM	.469**	−.232*	−.510**	−.131	.226**	.050	.415**	.501**	.128	−.151	−.273**	.574**	.720**	.839**

*Note*. MCI = mild cognitive impairment; MMSE = mini mental state examination; MoCA = montreal cognitive assessment; VF = verbal fluency; TMT = trail making test; IR = immediate recall; LC = learning curve; STM = short term memory; LTM = long term memory; EPT = everyday problem test.

a Refers to a dummy variable created to represent educational levels.

b Refers to a dummy variable created to represent health status.

* *p* < .05. **p < .001.

Linear regression modeling to predict EPT-G scores analysis yielded a statistically significant model (*p* < .000) that explained AR^2^ = 46.5% of the variance in EPT-G scores. Younger age, higher education, and being cognitively intact emerged as statistically significant independent predictors of higher EPT-G scores (see Table 4). The same model was examined with the time of completion for the EPT-G as the outcome variable, but the analysis did not yield a statistically significant model (*p* > .050).

**Table 4.** Regression Analysis Predicting EPT-G Scores Among the healthy and MCI Group.

	*B*	β	*p* Value
Age	−.257	−.241	.002
Gender _(male = 1, female = 2)_	−227	−.013	.862
Income	.001	.082	.278
Education^a^	2.267	.391	.000
Perceived health^b^	−.397	−.046	.480
Cognitive status^c^	3.740	.250	.001

*Note*. MCI = mild cognitive impairment.

a Refers to a dummy variable created to represent educational levels.

b Refers to a dummy variable created to represent health status.

c Mild cognitive impairment = 1, Cognitively intact = 2.

Stepwise regression models predicting EPT scores were conducted separately for the cognitively intact group and the MCI group separately to identify statistically significant predictors among the demographic and neuropsychological variables. The demographic variables (age, gender, income, and education) and health status were included in the first step, and all neuropsychological assessments (excluding MMSE and MoCA) were introduced in the second step. For the MCI group, the analysis yielded a statistically significant model (*p* < .000) that explained AR^2^ = 50.3% of the variance in EPT scores. Demographic variables independently explained the largest variance (AR^2^_change_ = 36.6%, *p* < .000) followed by performance in neuropsychological assessments (AR^2^_change_ = 23%, *p* < .005). In the first step of the model, age and education emerged as statistically significant independent predictors of EPT-G scores. The introduction of neuropsychological assessments in the second step result in age becoming not statistically significant independent predictors and gender becoming a statistically significant one. This suggests a moderation effect of CP in the relationship of age, and gender with EPT scores. Being a male, having higher education, and obtaining a higher score in immediate recall (IR) emerged as independent predictors of higher EPT scores in the final model (Table 5). For the cognitively healthy, the analysis did not yield a statistically significant model (*p* > .050).

**Table 5.** Stepwise Regression Analysis Predicting EPT-G Scores Among Participants of the MCI Group.

Step and variable	*B*	β	*p* Value	*R* ^2^_change_
Step 1				.363**
Age	−.349	−.276	.012	
Gender _(male_ _=_ _1, female_ _=_ _2)_	−.675	−.036	.773	
Income	.001	.077	.547	
Education^a^	2.634	.449	.000	
Perceived health^b^	.028	.003	.976	
Step 2				.230*
Age	−.200	−.158	.172	
Gender _(male_ _=_ _1, female_ _=_ _2)_	−5.744	−.310	.032	
Income	.000	.025	.835	
Education	1.802	.307	.007	
Perceived health	.521	.061	.539	
Verbal fluency	.187	.084	.416	
Trail making test—part A	−.007	−.014	.913	
Trail making test—part B	−.012	−.133	.317	
Immediate recall	1.580	.292	.013	
Learning curve	.036	.049	.730	
Short term memory	.198	.071	.691	
Long term memory	.442	.177	.285	

*Note*. MCI = mild cognitive impairment.

a Refers to a dummy variable created to represent educational levels.

b Refers to a dummy variable created to represent health status.

* *p* < .005. **p < .000.

## Discussion

This study was set out to translate, culturally adapt, and evaluate the psychometric properties of the Greek version of the EPT 28-item test. The adaptation and validation procedure followed a multi-step approach recommended by Geisinger (1994), including the review, the translation and adaption of the instrument, adapting the draft instrument based on the comments of the reviewers, pilot testing the instrument, and field testing the instrument. The evaluation of the psychometric properties for the EPT-G was based on the replication of the psychometric properties of the original EPT 28-items test. The correlations of the EPT-G instrument with age, education, and cognitive abilities were used as evidence of concurrent criterion-related validation.

The translation and cultural adaptation of the EPT-G was completed with a consensus among the panel of experts (i.e., two independent bilingual neuropsychologists, a Greek-English bilingual philologist of English literature and an English-Greek bilingual sociologist) that the instrument represents everyday tasks in Greece. The instrument was well received by the participants, and by the trainees who administrated the test. Notably, all the participants with suspected dementia and some of the participants with suspected MCI did not manage to complete the test. However, the evidence in this study indicates that the poor cognitive abilities among the dementia and MCI groups may account for the failure to complete the EPT-G, and not the comprehensibility of the instrument per se.

A minority of older women who did not drive found the material for the *driving tasks* in the EPT-G unfamiliar. The same claim was made by a minority of older women for the *financial issues* tasks (i.e., preparing the taxes sheet, balancing a bank account, applying for the automatic payment of electricity bills), stating for example: “*I have never done that. My husband takes care of such affairs.*” A minority of older men faced difficulties with the cooking recipes and nutritional guidelines tasks, stating: “*My wife usually does the cooking*.”

A minority of older women who did not drive found the material for the *driving tasks* in the EPT-G unfamiliar. The same claim was made by a minority of older women for the *financial issues* tasks (i.e., preparing the taxes sheet, balancing a bank account, applying for the automatic payment of electricity bills), stating for example: “*I have never done that. My husband takes care of such affairs.*” A minority of older men faced difficulties with the cooking recipes and nutritional guidelines tasks, stating: “*My wife usually does the cooking.*”

The emergence of unfamiliarity in some of the instruments’ tasks (i.e., driving, nutrition, cooking, and financial affairs) for a few participants is, to some extent, expected. The difference in the development of certain skills over the course of life may reflect socially constructed gender roles during the past century, were the division of labor and household duties was predominantly gendered. For example, Hsu (2015) argued that older women tend to be less financially literate, because they relied more on their spouses for these affairs in their lives. Similarly, women used to be underrepresented among active drivers in the past century, leading to fewer female older drivers to date (Bauer et al., 2003). Studies on male widowers have illustrated that a significant proportion of them have poor diets and a preference for ready-made meals (Hughes et al., 2004), presumably because of the poorer development of the pertinent skills over the course of their lives.

The EPT-G covers a variety of tasks with a range of difficulties. The overall difficulty of the tasks seems adequate for detecting subtle variations in EvF in cognitively healthy Greek older persons, as well as in those with suspected MC, avoiding ceiling or floor effects to a great extent. Better performance in the EPT-G was associated with younger age, higher educational levels, and better CP in well-established neuropsychological assessments of executive function, speed of processing, memory, and verbal fluency. This is consistent with the findings reported for the original instrument (Kimbler, 2013; Willis, 1993), replicating the patterns of correlations expected among CP and EvF assessment tools according to the literature on aging (Diehl et al., 2005). The EPT-G scores’ mean was within the range of scores reported in previous studies using the original instrument (depending on the age group *M_range_* = 13.03–17.08 in Kimbler, 2013).

Notably, the EPT-G may provide evidence for the comparative differentiation between those who are cognitively intact and those who have MCI. The small sample size of this study did not allow for the identification of cut off scores, and therefore no inference of cognitive status can be made at the individual level based only on the EPT-G, limiting this way the instruments’ applicability for clinical purposes. It is expected, however, that a population of older Greek persons with MCI will, on average, have lower scores in EPT-G than those who are cognitively intact. The evidence of this study also suggests that, among those with suspected MCI, poorer CP may lead to worse performance in the EPT-G. Hence, the EPT-G may be a sensitive tool that captures fluctuations in EvF among older adults who face mild cognitive impairments, differentiating them only comparatively from those who are cognitively intact.

The examination of EvF using the EPT-G retains some of the limitations of the original instrument. Since this instrument relies on language proficiency, its use for examining EvF is not recommended for non-native speakers (e.g., immigrants) or those who are illiterate. In this study, the categorization of participants into the cognitively healthy, MCI, or dementia groups was not based on an official diagnosis. Therefore, the interpretation of the study’s results in terms of cognitive status should be considered with caution. The small sample size of the group tested twice with this instrument may provide only preliminary evidence for the test-retest validity of the EPT-G instrument. The EPT-G seems to have a gender bias, with women scoring lower than men, although this bias seems to be attributable to gender differences in CP. Lastly, within the rapid development of modern societies, some of the tasks in the EPT-G may be, or may have become, outdated at the time of this publication.

### Conclusion

Despite these limitations, the evidence of this study indicates that the EPT-G is a well-adapted version of the original EPT 28-items measure of EvF that can be used for research purposes among Greek older adults who are cognitively intact or have MCI. In the Greek context, it can be used as an alternative to self-reported measures of EvF, especially when self-report biases are of great concern (Diehl et al., 2005). Future studies could examine whether the EPT-G can be used for clinical purposes, assisting health professionals in the diagnosis, and progression of MCI, where EvF impairments is a basic criterion with to discriminate between older adults who are cognitively healthy and those who have MCI.

## Supplemental Material

sj-docx-1-ggm-10.1177_23337214211027683 – Supplemental material for The Cultural Adaptation of the Everyday Problems Test—Greek Version: An Instrument to Examine Everyday Functioning Click here for additional data file. Supplemental material, sj-docx-1-ggm-10.1177_23337214211027683 for The Cultural Adaptation of the Everyday Problems Test—Greek Version: An Instrument to Examine Everyday Functioning by George Pavlidis, Stephanie Hatzifilalithis, Nikolaos Marwan Zawaher, Georgia Papaioannou, Eleni Giagkousiklidou and Ana B. Vivas in Gerontology and Geriatric Medicine

## References

[bibr1-23337214211027683] Aichberger M., C. Busch M., A. Reischies F., M Stroehle A. Heinz A. Rapp M., A. (2010). Effect of physical inactivity on cognitive performance after 2.5 years of follow up. Geropsychology, 23(1), 7–15. 10.1024/1662-9647/a000003

[bibr2-23337214211027683] Bauer M. J. Adler G. Kuskowski M. A. Rottunda S. (2003). The influence of age and gender on the driving patterns of older adults. Journal of Women & Aging, 15(4), 3–16. 10.1300/J074v15n04_02 14750586

[bibr3-23337214211027683] Bujang M. A. Baharum N. (2017). A simplified guide to determination of sample size requirements for estimating the value of intraclass correlation coefficient: A review. Archives of Orofacial Science, 12(1), 1–11.

[bibr4-23337214211027683] Bujang M. A. Sa’at N. Bakar T. M. I. T. A . (2017). Determination of minimum sample size requirement for multiple linear regression and analysis of covariance based on experimental and non-experimental studies. Epidemiology, Biostatistics and Public Health, 14(3), e12117-1–e12117-9. 10.2427/12117.

[bibr5-23337214211027683] Cantarella A. Borella E. Carretti B. Kliegel M. de Beni R. (2017). Benefits in tasks related to everyday life competences after a working memory training in older adults. International Journal of Geriatric Psychiatry, 32(1), 86–93. 10.1002/gps.4448 26968329

[bibr6-23337214211027683] Cherner M. (2010). Considerations in the cross-cultural assessment of functional abilities. In Marcotte T. D. Grant I. (Eds.), Neuropsychology of Everyday Functioning. The Guilford Press.

[bibr7-23337214211027683] Delis D., C. Kramer J., H. Kaplan E. Ober B., A. (1987). California Verbal Learning Test: Adult version. The Psychological Corporation.

[bibr8-23337214211027683] Diehl M. Marsiske M. Horgas A. L. Rosenberg A. Saczynski J. S. Willis S. L. (2005). The revised observed tasks of daily living. A performance-based assessment of everyday problem solving in older adults. Journal of Applied Gerontology, 24(3), 211–230. 10.1177/0733464804273772 18160968 PMC2153442

[bibr9-23337214211027683] Diehl M. Willis S. L. Schaie K. W. (1995) Everyday problem solving in older adults: Observational assessment and cognitive correlates. Psychology and Ageing, 10, 478–491. 10.1037//0882-7974.10.3.478 8527068

[bibr10-23337214211027683] Fountoulakis K. N. Tsolaki M. Chantzi H. Kazis A. (2000). Mini Mental State Examination (MMSE): A validation study in Greece. American Journal of Alzheimer’s Disease and other Dementias, 15, 342–345. 10.1177/153331750001500604

[bibr11-23337214211027683] Geisinger K. F. (1994). Cross-cultural normative assessment: Translation and adaptation issues influencing the normative interpretation of assessment instruments. Psychological Assessment, 6(4), 304. 10.1037/1040-3590.6.4.304

[bibr12-23337214211027683] Hambleton R. K. (2001). The next generation of the ITC test translation and adaptation guidelines. European Journal of Psychological Asessment, 17(3), 164–172. 10.1027/1015-5759.17.3.164

[bibr13-23337214211027683] Hering A. Kliegel M. Rendell P. G. Craik F. I. Rose N. S. (2018). Prospective memory is a key predictor of functional independence in older adults. Journal of the International Neuropsychological Society, 24(6), 640–645. 10.1017/S1355617718000152 29606153

[bibr14-23337214211027683] Hester R. L. Kinsella G. J. Ong B. McGregor J. (2005). Demographic influences on baseline and derived scores from the trail making test in healthy older Australian adults. The Clinical Neuropsychologist, 19, 45–54. 10.1080/13854040490524137 15814477

[bibr15-23337214211027683] Hsu J. W. (2015). Aging and strategic learning: The impact of spousal incentives on financial literacy. Journal of Human Resources, 51(4), 1063–1067. 10.3368/jhr.51.4.1014-6712R PMC5279946 28148971

[bibr16-23337214211027683] Hughes G. Bennett K. M. Hetherington M. M. (2004). Old and alone: Barriers to healthy eating in older men living on their own. Appetite, 43(3), 269–276. 10.1016/j.appet.2004.06.002 15527929

[bibr17-23337214211027683] Kelly M. E. Loughrey D. Lawlor B. A. Robertson I. H. Walsh C. Brennan S. (2014). The impact of cognitive training and mental stimulation on cognitive and everyday functioning of healthy older adults: A systematic review and meta-analysis. Ageing Research Reviews, 15, 28–43. 10.1016/j.arr.2014.02.004 24607830

[bibr18-23337214211027683] Kimbler K. J. (2013). Everyday problem solving and instrumental activities of daily living: Support for domain specificity. Behavioral Sciences, 3(1), 170–191. 10.3390/bs3010170 25379233 PMC4217610

[bibr19-23337214211027683] Knofczynski T. G. (2017). Sample sizes for predictive regression models and their relationship to correlation coefficients. Journal of Mathematical Sciences & Mathematics Education, 12(2), 12. http://www.msme.us/2017-2-2.pdf

[bibr20-23337214211027683] Kounti F. Tsolaki M. Kioseoglou G. (2006). Functional cognitive assessment scale (FUCAS): A new scale to assess executive cognitive function in daily life activities in patients with dementia and mild cognitive impairment. Human Psychopharmacological Clinical Experimentation, 21, 305–311. 10.1002/hup.772 16856217

[bibr21-23337214211027683] Larner A. J. (2018). Mini-mental state examination: Diagnostic test accuracy study in primary care referrals. Neurodegenerative Disease Management, 8(5), 301–305. 10.2217/nmt-2018-0018 30223710

[bibr22-23337214211027683] Lawton M. P. Brody E. M. (1969). Assessment of older people: Self-maintaining and instrumental activities of daily living. Gerontologist, 9, 179–186. 5349366

[bibr23-23337214211027683] Lindbergh C. A. Dishman R. K. Miller L. S. (2016). Functional disability in mild cognitive impairment: A systematic review and meta-analysis. Neuropsychology Review, 26(2), 129–159. 10.1007/s11065-016-9321-5 27393566

[bibr24-23337214211027683] Lumley T. Diehr P. Emerson S. Chen L. (2002). The importance of the normality assumption in large public health data sets. Annual Review of Public Health, 23(1), 151–169. https://doi.org/23.100901.140546 10.1146/annurev.publhealth.23.100901.140546 11910059

[bibr25-23337214211027683] Marcotte T. D. Scott J. C. Kamat R. M Heaton R. K. (2010). Neuropsychology and the prediction of everyday functionality. In Marcotte T. D. Grant I. (Eds.), Neuropsychology of Everyday Functioning. The Guilford Press.

[bibr26-23337214211027683] Martyr A. Clare L. (2012). Executive function and activities of daily living in Alzheimer’s disease: A correlational meta-analysis. Dementia Geriatric Cognitive Disorders, 33(2–3), 189–203. 10.1159/000338233 22572810

[bibr27-23337214211027683] Mcalister C. Schmitter-Edgecombe M. Lamb R. (2016). Examination of variables that may affect the relationship between cognition and functional status in individuals with mild cognitive impairment: A meta-analysis. Archives of Clinical Neuropsychology, 31(2), 123–147. 10.1093/arclin/acv089 26743326 PMC4758380

[bibr28-23337214211027683] Moore D. J. Palmer B. M. Patterson T. L. Jeste D.V. (2007). A review of performance-based measures of functional living skills. Journal of Psychiatric Research, 41, 97–118. 16360706 10.1016/j.jpsychires.2005.10.008

[bibr29-23337214211027683] Nordin S. Elf M. McKee K. Wijk H. (2015). Assessing the physical environment of older people’s residential care facilities: Development of the Swedish version of the Sheffield Care Environment Assessment Matrix (S-SCEAM). BMC Geriatrics, 15(1), 3. http://www.biomedcentral.com/1471-2318/15/3 25563507 10.1186/1471-2318-15-3 PMC4323237

[bibr30-23337214211027683] Peel N. Bartlett H. McClure R. (2004). Healthy ageing: How is it defined and measured? Australasian Journal on Ageing, 23(3), 115–119.

[bibr31-23337214211027683] Petersen R. C. (2004). Mild cognitive impairment as a diagnostic entity. Journal of Internal Medicine, 256, 183–194. 15324362 10.1111/j.1365-2796.2004.01388.x

[bibr32-23337214211027683] Petersen R. C. Lopez O. Armstrong M. J. Getchius T. S. Ganguli M. Gloss D. Gronseth G. S. Marson D. Pringsheim T. Day G. S. Sager M. Stevens J. Rae-Grant A. (2018). Practice guideline update summary: Mild cognitive impairment: Report of the guideline development, dissemination, and implementation Subcommittee of the American Academy of Neurology. Neurology, 90(3), 126–135. 10.1212/WNL.0000000000004826 29282327 PMC5772157

[bibr33-23337214211027683] Poptsi E. Moraitou D. Eleftheriou M. Kounti-Zafeiropoulou F. Papasozomenou C. Agogiatou C. Bakoglidou E. Batsila G. Liapi D. Markou N. Nikolaidou E. Ouzouni F. Soumpourou A. Vasiloglou M. Tsolaki M. (2019). Normative data for the montreal cognitive assessment in Greek older adults with subjective cognitive decline, mild cognitive impairment and dementia. Journal of Geriatric Psychiatry and Neurology, 32(5), 265–274. https://doi.org/10.1177%2F0891988719853046 31159629 10.1177/0891988719853046

[bibr34-23337214211027683] Ramanan S. Bertoux M. Flanagan E. Irish M. Piguet O. Hodges J. R. Hornberger M. (2017). Longitudinal executive function and episodic memory profiles in behavioral-variant frontotemporal dementia and Alzheimer’s disease. Journal of the International Neuropsychological Society, 23(1), 34–43. 10.1017/S1355617716000837 27751195

[bibr35-23337214211027683] Rodin J. McAvay G. (1992). Determinants of change in perceived health in a longitudinal study of older adults. Journal of Gerontology, 47(6), 373–384. 10.1093/geronj/47.6.p373 1430859

[bibr36-23337214211027683] Royall D. R. Lauterbach E. C. Malloy P. Coburn K. L. Blak K. J. (2007). The cognitive correlates of functional status: A review from the committee on research of the American Neuropsychiatric Association. Journal of Neuropsychiatry and Clinical Neuroscience, 19, 249–265. 10.1176/jnp.2007.19.3.249 17827410

[bibr37-23337214211027683] Scheel-Hincke L. L. Möller S. Lindahl-Jacobsen R. Jeune B. Ahrenfeldt L. J. (2020). Cross-national comparison of sex differences in ADL and IADL in Europe: Findings from SHARE. European Journal of Ageing, 17(1), 69–79. 10.1007/s10433-019-00524-y 32158373 PMC7040124

[bibr38-23337214211027683] Schmitter-Edgecombe M. Farias S. T. (2018). Aging and everyday functioning: Measurement, correlates, and future directions. In Smith G. E. Farias S. T. (Eds.), APA handbook of dementia. American Psychological Association.

[bibr39-23337214211027683] Tomaszewski-Farias S. Schmitter-Edgecombe M. Weakley A. Harvey D. Denny K. G. Barba C. Gravano J. T. Giovannetti T. Willis S. (2018). Compensation strategies in older adults: Association with cognition and everyday function. American Journal of Alzheimer’s Disease & Other Dementias, 33(3), 184–191. https://doi.org/10.1177%2F1533317517753361 10.1177/1533317517753361 PMC10852491 29357670

[bibr40-23337214211027683] van Harten A. C. Mielke M. M. Swenson-Dravis D. M. Hagen C. E. Edwards K. K. Roberts R. O. Geda Y. E. Knopman D. S. Petersen R. C . (2018). Subjective cognitive decline and risk of MCI: The Mayo Clinic Study of aging. Neurology, 91(4), 300–312. https://doi.org/10.1212%2FWNL.0000000000005863 10.1212/WNL.0000000000005863 PMC6070384 29959257

[bibr41-23337214211027683] Vlachos G. S. Kosmidis M. H. Yannakoulia M. Dardiotis E. Hadjigeorgiou G. Sakka P. , et al. (2020). Prevalence of mild cognitive impairment in the elderly population in Greece: Results from the HELIAD study. Alzheimer Disease & Associated Disorders, 34(2), 156–162. doi: 10.1097/WAD.0000000000000361. 31913961

[bibr42-23337214211027683] Vlachou C. H. Kosmidou M. H. (2002). Η ελληνική δοκιμασία οπτικο-νοητικής ιχνηλάτησης: Προκαταρτικές νόρμες για κλινική και ερευνητική εφαρμογή. Psychology, 9(3), 336–352.

[bibr43-23337214211027683] Vlachou C. H. Kosmidis M. H. Dardagani A. Tsotsi S. Giannakou M. Giazkoulidou A. Zervoudakis E. Pontikakis N. (2012). Development of the Greek verbal learning test: Reliability, construct validity, and normative standards. Archives of Clinical Neuropsychology, 28, 52–64. 10.1093/arclin/acs099 23179043

[bibr44-23337214211027683] Willis S. L. (1990). Everyday problems test. Pennsylvania State University.

[bibr45-23337214211027683] Willis S.L. (1993). Test manual for everyday problem test for cognitvely challenged elderly. Pennsylvania State University. https://sls.psychiatry.uw.edu/wp-content/uploads/2020/03/Manual-EPT-Cgn-Chal-Elderly-copy.pdf

[bibr46-23337214211027683] Willis S. L. Marsiske M. (1993). Manual for the everyday problems test. Department of Human Development and Family Studies, Pennsylvania State University.

[bibr47-23337214211027683] Willis S. L. Tennstedt S. L. Marsiske M. Ball K. Elias J. Koepke K. M. Morris J. N. Rebok G. W. Unverzagt F. W. Stoddard A. M. Wright E. ; ACTIVE Study Group. (2006). Long-term effects of cognitive training on everyday functional outcomes in older adults. Journal of the American Medical Association, 296, 2805–2814. 17179457 10.1001/jama.296.23.2805 PMC2910591

