# Supplementary material for: The Cultural Adaptation of the Everyday Problems Test—Greek Version: An Instrument to Examine Everyday Functioning
Source: Gerontol Geriatr Med. 2021 Jul 5;7:23337214211027683. doi: 10.1177/23337214211027683 (PMC8261850; doi:10.1177/23337214211027683)
Supplement: sj-docx-1-ggm-10.1177_23337214211027683 – Supplemental material for The Cultural Adaptation of the Everyday Problems Test—Greek Version: An Instrument to Examine Everyday Functioning [file sj-docx-1-ggm-10.1177_23337214211027683.docx]

**Appendix I**

Translation and adaptation process from the Everyday Problems Test (28 items) to Everyday Problems Test – Greek version

| Step | Process | Outcome |
| --- | --- | --- |
| 1. Adaptation -translation of the instrument | - Substitution of metrics and adaptation of content - Translation – back-translation | - The Everyday Problems Test – Greek version, 28 items. |
| 1. Revision of the translated and adapted version based on reviewers’ comments | - Assessing the conceptual equivalence of the adjusted items and the quality of the translation - Assessing the linguistic appropriateness | - No further modifications were proposed for the instrument. |
| 1. Pilot testing of the instrument on 13 older individuals | - Examining the understandability of instructions and of items | - The test was well received and comprehended. - No changes were proposed |
| 1. Field testing of the instrument | - Internal consistency analysis - Inter-rater reliability analysis - Test-retest reliability | - High internal consistency - No statistically significant differences among examiners - High test-retest reliability |
| 1. Validation: concurrent criterion validity | - Pearson correlation coefficient analysis with age, education, and cognitive measures | - The EPT-G was correlated statistically significant with age, education, and measures of executive function |
| 1. Developing a manual and other documentation for the users of the assessment | - Translating the scoring guide of the EPT 28-items - Adapting the manual according to the modified items | - Scoring manual for the EPT-G |
| 1. Training users – collecting reactions from users | - Training users for 5-15 hours - Collecting comments from users | - No changes were proposed for the instrument or the scoring manual |
